# HeartMate 3 Left Ventricular Assist Device Implantation in a Pediatric Patient With Limb-Girdle Muscular Dystrophy

**DOI:** 10.31486/toj.24.0109

**Published:** 2025

**Authors:** Christopher M. Zumwalt, Katerina Boucek, Dennis A. Wells

**Affiliations:** ^1^ Department of Surgery, Ochsner Clinic Foundation, New Orleans, LA; ^2^ Division of Pediatric Cardiology, Ochsner Children's Hospital, Ochsner Clinic Foundation, New Orleans, LA; ^3^ Division of Pediatric and Congenital Cardiac Surgery, Ochsner Children's Hospital, Ochsner Clinic Foundation, New Orleans, LA; ^4^ The University of Queensland Medical School, Ochsner Clinical School, New Orleans, LA

**Keywords:** *Cardiomyopathies*, *heart-assist devices*, *muscular dystrophies*, *pediatrics*

## Abstract

**Background:**

The development of cardiac complications is common in patients with muscular dystrophy. However, advanced heart failure therapies such as implantation of durable ventricular assist devices and transplant are infrequently used in patients who develop cardiomyopathy, often because of comorbid impairments in mobility and respiratory function.

**Case Report:**

A 16-year-old male with limb-girdle muscular dystrophy type R4 presented with chronic decompensated heart failure. Recent worsening of his respiratory function and mobility were confounded by severe heart failure. In addition to our core advanced heart failure team, multidisciplinary assessment preoperatively included Neurology, Pulmonology, Genetics, and Physical Medicine and Rehabilitation. The patient underwent implantation of a HeartMate 3 left ventricular assist device and had an uneventful postoperative course. After intensive inpatient physical and occupational therapy, he was discharged home on postoperative day 16 with minimal residual heart failure symptoms and plans to continue robust outpatient physical therapy.

**Conclusion:**

Patients with muscular dystrophy often have cardiac involvement; however, certain subtypes of muscular dystrophy are associated with an earlier presentation of severe life-limiting cardiomyopathy. Pediatric patients with muscular dystrophy should be considered for advanced heart failure therapies such as implantation of a durable left ventricular assist device at an appropriate center. Carefully selected patients may experience substantial improvements in their quality of life. Given the variable disease progression and life expectancy of patients with subtypes of muscular dystrophy, a thorough assessment by a multidisciplinary team is critical.

## INTRODUCTION

Muscular dystrophies are a heterogeneous group of neuromuscular disorders that are characterized by a progressive decline in skeletal muscle strength and often by a decline in respiratory function as well. The heart is commonly impacted with cardiomyopathy of varying severity.^1^ Duchenne and Becker muscular dystrophies, X-linked recessive disorders involving mutation of the dystrophin gene that results in either absent or diminished dystrophin protein production, respectively, make up the majority of cases, and both types are associated with cardiac involvement.^1^ Advanced heart failure therapies, including transplant and ventricular assist devices (VADs), have been used in such patients.^2-4^

Limb-girdle muscular dystrophies (LGMD) are rarer than the Duchenne and Becker types and include a number of subtypes with varying phenotypes and symptom severity, but all are commonly linked by skeletal muscle weakness typically developing earliest in the shoulders and hips.^5,6^

Our patient has a particularly rare and severe form of LGMD type R4, now referenced as LGMDR4, but also known as type 2E LGMD or beta-sarcoglycanopathy. LGMDR4 is an autosomal recessive disorder that causes a deficiency of the protein beta-sarcoglycan typically found in skeletal muscle and cardiac myocytes. LGMDR4 is frequently associated with cardiomyopathy that can be severe and in the form of hypertrophic or dilated cardiomyopathy.^6,7^ Cardiac disease is the most common cause of death in patients with LGMDR4; patients with cardiac involvement may have a life expectancy of only 20 to 30 years,^7^ although prognosis is difficult given the small number of patients with LGMD (prevalence rates are estimated at 0.8 per million).^8^

To our knowledge, our case is only the third report of left ventricular assist device (LVAD) implantation in a pediatric patient with LGMD, and he is the only patient discharged home on the device.^9^

## CASE REPORT

A 16-year-old male with muscular dystrophy and 2 years of known cardiomyopathy presented to another facility with decompensated heart failure. The presentation was his second heart failure admission in 3 months despite optimal medical therapy as an outpatient, including home losartan (50 mg daily), furosemide (20 mg daily), carvedilol (6.25 mg twice daily), and aspirin (81 mg daily). He had experienced progressive dyspnea and fatigue for a few weeks. Based on echocardiography, the patient's ejection fraction had declined from 25% to 30% 3 months prior to approximately 10%. He was treated with inotropic support with milrinone and aggressive diuresis with intravenous furosemide, resulting in a 9 kg weight loss over the course of 1 week prior to transfer to our facility for consideration of advanced therapies including an LVAD.

The patient was evaluated by our pediatric advanced heart failure team that includes a social worker, psychologist, physical and occupational therapist, nutritionist, and palliative medicine physician. Given the patient's LGMD, Pediatric Neurology, Pulmonology, Genetics, and Physical Medicine and Rehabilitation were also consulted. Our Neurology and Genetics colleagues provided important evaluation and prognostic information regarding the patient's LGMD, Pulmonology assessed his baseline respiratory function and provided recommendations to maximize his respiratory function, and Physical Medicine and Rehabilitation formulated and implemented a rigorous rehabilitation program.

The most important factors to determine prior to considering the patient's suitability for implantation of a durable VAD were his long-term prognosis related to progression of the LGMD and the severity of his current levels of impaired mobility and respiratory function. During the prior year, the patient had experienced progressive limitations in mobility, necessitating the use of a walker and frequent use of a wheelchair for daily activities. His respiratory symptoms were confounded by his severe heart failure, but he had not required positive pressure ventilation, and his pulmonary function tests demonstrated a moderately restrictive pattern. No question existed regarding the short-term benefits the patient would receive from a VAD, but it was important to determine that a reasonable expectation existed for a legitimate long-term benefit. In the short term, the patient was not considered a candidate for cardiac transplant primarily because of the degree of his mobility impairment and the rate of progression. The possibility for assessment for transplant candidacy would remain open in the future once the patient was well enough and an assessment of his limits related to muscular dystrophy could be more clearly distinguished from the sequelae of his heart failure.

The multidisciplinary team decided to proceed with LVAD implantation as the device would increase the patient's cardiorespiratory function and improve his quality of life. The implant would be performed as a bridge to decision for cardiac transplant. A comprehensive postoperative rehabilitation plan was prepared.

Cardiac catheterization was performed prior to implantation to further assess the severity of the patient's at-least-moderate right ventricular dysfunction that was demonstrated on echocardiogram. Echocardiogram demonstrated normal valvular structures with moderate mitral insufficiency. At the time of catheterization, the patient was administered 0.5 μg/kg/min of milrinone and 0.02 μg/kg/min of epinephrine. His heart rate was 124 beats per minute, and his blood pressure was 125/78 mm Hg. The catheterization demonstrated dilated cardiomyopathy with low cardiac output and moderate pulmonary hypertension but indicated a low risk for the necessity of temporary right ventricular support as detailed by the following hemodynamic data: cardiac index 1.8 L/min/m^2^ (body surface area 1.56 m^2^; weight 46.8 kg); cardiac output 2.8 L/min; right atrial pressure 12 mm Hg; right ventricular pressure 62/12 mm Hg; pulmonary artery pulsatility index 4.2; right ventricular stroke work index 6.35 g/m/beat/m^2^; pulmonary artery pressure 62/34 mm Hg (mean 44 mm Hg); pulmonary capillary wedge pressure 38 mm Hg; and pulmonary vascular resistance of 3 Wood units. A CardioMEMS device (Abbott Cardiovascular) was placed at the time of cardiac catheterization to allow outpatient monitoring of the patient's pulmonary pressures and help fine-tune his VAD settings and diuretic regimen postoperatively.

The patient was determined to be an appropriate surgical candidate, and on hospital day 5, he underwent implantation of a HeartMate 3 LVAD (Abbott Cardiovascular). The operation was uncomplicated, and the patient was weaned from bypass on typical inotropic support and inhaled nitric oxide for right ventricular support. His postoperative course was unremarkable. He was extubated on postoperative day 1 and did quite well with the expected additional support needed from Physical and Occupational Therapy that worked aggressively to mobilize him early and often with inpatient physical rehabilitation. He was weaned from all inotropic and inhaled nitric oxide support by postoperative day 4. Echocardiogram demonstrated normal right ventricular size with moderately decreased systolic function. He was discharged on postoperative day 16 in substantially improved cardiorespiratory condition, although he was still using a walker and wheelchair as he had been prior to surgery.

Upon discharge, the patient was enrolled in cardiac rehabilitation and outpatient physical and occupational therapy, and he was scheduled for regular clinic visits. At follow-up 3 months after discharge, he reported substantially increased endurance. He was able to complete activities of daily living independently and was no longer using the wheelchair.

## DISCUSSION

We are optimistic that our case will encourage clinicians at centers with the appropriate resources to keep an open mind regarding what advanced heart failure therapies can be offered to carefully selected pediatric patients, such as patients with muscular dystrophy whose cardiac dysfunction progression is expected to outpace their skeletal and respiratory dysfunction progression and who meet VAD selection criteria.

A 2023 report from the Advanced Cardiac Therapies Improving Outcomes Network (ACTION) registry, which includes more than 50 participating centers, demonstrated that until 2020, only 12 pediatric centers had implanted a VAD in patients with any form of muscular dystrophy.^9^ The total number of patients implanted was just 18, with the majority having Duchenne or Becker muscular dystrophies; only 11 were pediatric patients. Only 2 cases involved patients with LGMD, both of whom underwent cardiac transplant prior to discharge from the hospital.^9^

The ACTION report demonstrates the rarity of VAD implantation and the small number of centers performing the procedure. Despite the comorbidities of patients with muscular dystrophy, the outcomes of the cohort were overall positive and comparable to the general VAD population.^9^ Durable VAD implantation in patients with muscular dystrophy, particularly those with substantial impairment of their mobility and respiratory function, must be considered on a case-by-case basis. Multidisciplinary team evaluation is essential. Prognosis may be difficult to predict, but colleagues from Neurology, Pulmonology, Genetics, and Physical Medicine and Rehabilitation can be invaluable in the evaluation process to help guide the team's decision-making. Moreover, in cases of durable VAD implantation, appropriate resources must be identified for ongoing rehabilitation after discharge.

## CONCLUSION

Pediatric patients with muscular dystrophy should be considered for advanced heart failure therapies including durable LVAD or transplant at appropriate centers. The benefits to carefully selected patients with early progression of cardiac dysfunction can be substantial. Given variable disease progression and life expectancy among patients with subtypes of muscular dystrophies, a thorough assessment by a multidisciplinary team is imperative for selecting and optimizing the right patients.

## Notes

*This article meets the Accreditation Council for Graduate Medical Education and the American Board of Medical Specialties Maintenance of Certification competencies for Patient Care and Medical Knowledge.*
